# WSe_2_/g-C_3_N_4_ for an In Situ Photocatalytic Fenton-like System in Phenol Degradation

**DOI:** 10.3390/nano12183089

**Published:** 2022-09-06

**Authors:** Li Tan, Yiming Chen, Didi Li, Shaobin Wang, Zhimin Ao

**Affiliations:** 1 Guangdong-Hong Kong-Macao Joint Laboratory for Contaminants Exposure and Health, Guangzhou Key Laboratory Environmental Catalysis and Pollution Control, Institute of Environmental Health and Pollution Control, Guangdong University of Technology, Guangzhou 510006, China; 2 Guangdong Key Laboratory of Environmental Catalysis and Health Risk Control, Key Laboratory for City Cluster Environmental Safety and Green Development of the Ministry of Education, School of Environmental Science and Engineering, Guangdong University of Technology, Guangzhou 510006, China; 3 School of Chemical Engineering and Advanced Materials, The University of Adelaide, Adelaide, SA 5005, Australia; 4 Advanced Interdisciplinary Institute of Environment and Ecology, Beijing Normal University, Zhuhai 519087, China

**Keywords:** WSe_2_/g-C_3_N_4_, visible light, in situ photo-Fenton, phenol degradation

## Abstract

An in situ photo-Fenton system can continuously generate H_2_O_2_ by photocatalysis, activating H_2_O_2_ in situ to form strong oxidizing ·OH radicals and degrading organic pollutants. A WSe_2_/g-C_3_N_4_ composite catalyst with WSe_2_ as a co-catalyst was successfully synthesized in this work and used for in situ photo-Fenton oxidation. The WSe_2_/g-C_3_N_4_ composite with 7% loading of WSe_2_ (CNW2) has H_2_O_2_ production of 35.04 μmol/L, which is fourteen times higher than pure g-C_3_N_4_. The degradation efficiency of CNW2 for phenol reached 67%. By constructing an in situ Fenton-system, the phenol degradation rate could be further enhanced to 90%. WSe_2_ can enhance the catalytic activity of CNW2 by increasing electron mobility and inhibiting the recombination of photogenerated electron–hole pairs. Moreover, the addition of Fe^2+^ activates the generated H_2_O_2_, thus increasing the amount of strong oxidative ·OH radicals for the degradation of phenol. Overall, CNW2 is a promising novel material with a high H_2_O_2_ yield and can directly degrade organic pollutants using an in situ photo-Fenton reaction.

## 1. Introduction

Phenol is a major pollutant in industrial wastewater such as oil refineries, petrochemical plants, coking plants, and phenolic resin plants [1]. Phenol may be produced in certain agricultural products and animal manure as well [2]. Phenol is a highly toxic organic pollutant that poses a health threat to humans and biota [3]. In recent decades, advanced oxidation processes (AOPs) have proven attractive for wastewater treatment [4]. Thus far, AOPs have been successfully applied to the degradation of persistent organic pollutants, and can convert toxic organic pollutants into easily degradable low molecular weight metabolites [5]. As a typical kind of AOP, Fenton reaction can take advantage of Fe^2+^ to activate hydrogen peroxide (H_2_O_2_) in order to form ·OH radicals, as shown in Equation (1).

(1) $$Fe^{2+}+H_{2}O_{2}\to Fe^{3+}+\cdot OH+OH^{-}$$

·OH can be used as an unselective reactive oxygen species (ROS) to degrade most organic pollutants in aqueous solutions [6]. Early in 1992, Zepp et al. [7] studied the kinetic mechanism of organic pollutants removal, including oxalate, citrate, and phosphate complexes through a photo-Fenton system constructed by Fe^2+^. This has been the foundation for subsequent research on the photo-Fenton reaction in pollutant removal. The traditional photo-Fenton reaction requires the addition of H_2_O_2_, which increases costs and limits its practical application. In contrast to the conventional Fenton reaction, which requires the addition of H_2_O_2_, an in situ photo-Fenton system can continuously generate H_2_O_2_ by photocatalysis and activate H_2_O_2_ in situ to form strong oxidizing ·OH radicals, accelerating the degradation of various pollutants [8,9]. In the in situ photo-Fenton system, the role of the catalyst is very important, as it is related to the production of H_2_O_2_ and the degradation of pollutants. Therefore, developing a photocatalyst to construct an in situ photo-Fenton system with high reactivity to produce H_2_O_2_ is critical for the degradation of pollutants.

Among various visible light-responsive materials, graphitized carbon nitride (g-C_3_N_4_) has attracted much attention because of its effective visible light absorption, suitable conduction band edge, high stability, and excellent environmental friendliness [10,11,12,13]. In 2014, Shiraishi et al. [14] discovered that g-C_3_N_4_ can be used as a photocatalyst for H_2_O_2_ production thanks to its high selectivity in ethanol/water mixed solutions when exposed to visible light (λ > 420 nm). Many subsequent studies have confirmed these findings [15,16,17]. However, bulk g-C_3_N_4_ usually exhibits poor photocatalytic activity owing to the low separation and transfer efficiency of photo-generated carriers [18]. Therefore, a series of modifications have been made to g-C_3_N_4_ to improve its photocatalytic activity.

Developing a precious metal-free co-catalyst with g-C_3_N_4_ as a composite material is a potential modification approach [19,20]. In recent years, transition metal dichalcogenides (TMDs) have attracted wide attention due to their unique layered structure, high stability, and excellent electronic and electro-optical properties [21,22]. Tungsten selenite (WSe_2_) is a type of TMD that is widely used in energy and environmental areas, including in photodetectors [23], field-effect transistors [24,25], photocatalytic CO_2_ reduction reactions (CO_2_RR) [26], and water splitting [27]. Guo et al. [28] found that using WSe_2_ nanosheets as a co-catalyst significantly increased the rate of photocatalytic H_2_ production by Zn_0.1_Cd_0.9_S nanorods. With visible light as a driver, the generation rate of H_2_ was 147.32 mmol h^−1^ g_cat_^−1^, which is eleven times the initial ate of Zn_0.1_Cd_0.9_S. Similarly, Lin et al. [29] reported a floating plate photocatalytic system with WSe_2_ as the co-catalyst which had an H_2_ evolution rate of 64.85 mmol h^−1^ g^−1^. These results suggest that WSe_2_ is an effective co-catalyst that can act as an electron absorber to accelerate the separation of space carriers, thereby enhancing the performance of the photocatalytic reaction [30]. Wang et al. [30] synthesized a WSe_2_/g-C_3_N_4_ photocatalyst which showed high H_2_O_2_ production and high bacterial inactivation efficiency. However, research on WSe_2_ nanosheets as co-catalysts for photocatalytic organic pollutant removal is rarely reported.

In this work, WSe_2_/g-C_3_N_4_ composite catalysts with WSe_2_ as a co-catalyst were successfully synthesized by a hydrothermal method combined with a calcination method. The photocatalytic H_2_O_2_ production and the degradation of phenol with pure g-C_3_N_4_ and the WSe_2_/g-C_3_N_4_ composite catalysts were tested in pure water under visible light. At the same time, Fe^2+^ was added to create an in situ optical Fenton system for phenol degradation. Coumarin was used as a trap to detect the ·OH produced by the photocatalytic reaction. This work shows that the WSe_2_/g-C_3_N_4_ composite is a potential photocatalyst that can produce H_2_O_2_ in pure water and be directly used in an in situ photo-Fenton reaction to degrade organic pollutants.

## 2. Materials and Methods

### 2.1. Chemicals and Materials

Melamine (C_3_H_6_N_6_ 99%, analytically pure) was obtained from Shanghai Macklin Biochemical Technology Co., Ltd., Shanghai, China. Selenium powder, sodium tungstate dihydrate (Na_2_WO_6_·2H_2_O 99.5%, analytically pure), sodium borohydride (NaBH_4_, analytically pure), tungsten selenide (WSe_2_ 99%, analytically pure), ferrous sulfate heptahydrate (FeSO_4_·7H_2_O > 99%, analytically pure), phenol (C_6_H_6_O, analytically pure), horseradish peroxidase (POD, RZ > 3.0), N,N-diethyl-p-phenylenediamine sulfate salt (DPD), and coumarin (C_9_H_6_O_2_ 99%, analytically pure) were all provided by Shanghai Aladdin Biochemical Technology Co., Ltd., Shanghai, China. N,N-dimethylformamide (C_3_H_7_NO, analytically pure) was purchased from Guangzhou Chemical Reagent Factory, Guangzhou, China. Ultra-pure water was used in all experiments.

### 2.2. Synthesis

**Preparation of g-C_3_N_4_**: The original g-C_3_N_4_ (labeled as PCN) was prepared by thermally polymerizing melamine [31]. The specific experimental operation is as follows: 10 g of C_3_H_6_N_6_ powder was placed into a crucible, then the crucible was heated under 520 °C for 2 h in a box furnace. After the sample was naturally cooled, a yellow solid was obtained and was collected after grinding.

**Preparation of WSe_2_/g-C_3_N_4_:** According to the previous method [30], several WSe_2_/g-C_3_N_4_ composite materials with different ratios were synthesized by the combination of a hydrothermal and calcination method. The specific experimental operation was as follows: a mixture of selenium powder (Se, 57.7 mg, 0.731 mmol) and sodium tungstate dihydrate (Na_2_WO_6_·2H_2_O, 120.75 mg, 0.366 mmol) was added to a beaker containing N, N-dimethylformamide (DMF, 60 mL). Then, 100 mg of sodium borohydride (NaBH_4_) was slowly added under constant stirring, and the mixture solution was continuously stirred for 2 h. After that, 2.5 g of PCN was added to the above mixed solution and stirred for 1 h. After the stirring, the solution was transferred into a closed autoclave with a volume of 100 mL and went subjected to heat treatment at 240 °C for 24 h. The solution was cooled to room temperature and washed with ultrapure water and absolute ethanol several times, then dried under vacuum at 60 °C for 24 h. Finally, the obtained solid was ground into powder, which was calcined and annealed in a tube furnace at 300 °C for 5 h under an argon atmosphere. The WSe_2_/g-C_3_N_4_ material with a WSe_2_ loading of 5% was named as CNW1. To determine the WSe_2_/g-C_3_N_4_ material with the best photocatalytic performance, other samples with 7%, 10%, and 14% loadings of WSe_2_ were prepared. These materials were marked as CNW2, CNW3, and CNW4, respectively.

**Preparation of WSe_2_:** A mixture of Se powder (315.6 mg, 4 mmol) and Na_2_WO_6_·2H_2_O (659.6 mg, 2 mmol) was added to a beaker containing 60 mL DMF while stirring. Then, 100 mg NaBH_4_ was slowly added into the mixed solution and stirred continuously for 3 h. The rest of the operation was the same as the preparation method used for WSe_2_/g-C_3_N_4_.

### 2.3. Characterizations

X-ray diffraction (XRD) patterns of the synthesized materials were obtained from an X-ray diffractometer with a Cu-Kα radiation source (λ = 1.5218 Å) (D8 ADVANCE, Bruker Inc., Saarbrucken, Germany). Transmission electron microscopy (TEM) images were examined on a Talos F200S (FEI, Thermo, Inc., Czech Republic) field-emission transmission electron microscope operated at 200 kV. Field emission scanning electron microscopy (FESEM) with energy dispersive X-ray spectroscopy (EDX) elemental mapping images were taken by a field-emission electron microscope (SU8220, Hitachi Ltd., Tokyo, Japan) with an acceleration voltage of 15 kV. X-ray photoelectron spectroscopy (XPS) spectra were obtained from a Escalab 250Xi (Thermo Fisher Scientific, Inc., Waltham, MA, USA) spectrometer with Al Kα radiation. Photoluminescence (PL) spectra were obtained using an FS5 (Edinburgh Inc., Edinburgh, UK) fluorescence spectrophotometer under 380 nm excitation. UV–vis diffuse reflectance spectra (UV–vis DRS) were acquired on a Cary 300 spectrophotometer (Agilent Technologies Inc., Palo Alto, CA, USA).

### 2.4. Photocatalytic Performance

**Photocatalytic production of H_2_O_2_**: This experiment was carried out in a PCX50B Discover multi-channel photocatalytic system (5 W, λ > 420 nm, Perfectlight Technology Co., Ltd., Beijing, China). Typically, a catalyst sample (30 mg) was added to a reactor containing 30 mL of pure water. Before being exposed to light, a dark adsorption treatment was carried out for 30 min. Then, the light was turned on and the reaction proceeded for 2 h. During the photocatalytic reaction, 1 mL of the solution was collected every 20 min and filtered through a polytetrafluoroethylene (PES) millipore filter (0.22 μm) to remove the photocatalyst powders. Finally, the amount of H_2_O_2_ produced by photocatalysis was determined by the DPD-POD method [32]. The specific method was as follows: 1 mL of sample aliquots were mixed with 1.12 mL water, 0.4 mL phosphate buffered solution, 0.05 mL POD (1 mg/mL^−1^), and 0.05 mL DPD (10 mg/mL). Vigorous stirring was maintained for 1 min, then the absorbance of the mixed liquid was measured at 551 nm on a multifunctional microplate reader (Varioskan LUX, Thermo Fisher Scientific, Inc., Waltham, MA, USA). To ensure the accuracy of the experiment, the absorbance of H_2_O_2_ was measured three times.

**Photocatalytic degradation of phenol**: In this experiment, phenol with a concentration of 10 ppm (10 mg/L) was used as the target pollutant. The phenol degradation performance of the prepared photocatalysts was tested under the visible light irradiation of the PCX50B Discover multi-channel photocatalytic system. A catalyst sample (30 mg) was added to a reactor with 30 mL of phenol solution (10 ppm). Before exposure to light, a dark adsorption treatment was carried out for 30 min. Then, the light was turned on and the reaction proceeded for 6 h. During the photocatalytic reaction process, water samples were taken every 1 h. The solution was transferred into a high-performance liquid chromatography (HPLC, Eclassical 3100, Elite Analytical Instrument Co., Ltd., Dalian, China) vial, and the concentration of phenol was analyzed and determined by HPLC equipment with a UV detector. Methanol and ultra-pure water (40:60) were employed as the mobile phases at a flow rate of 0.8 mL min^−1^ and the wavelength of the detector was set at 270 nm. The degradation rate of phenol can be expressed by Equation (2)

(2) $$D=1-\left(\frac{C}{C_{0}}\right)\times 100\%$$

where *C* and *C*_0_ represent the concentrations of phenol at a specific interval and the initial time, respectively [33].

**In situ photo-Fenton degradation of phenol**: The H_2_O_2_ generated by photocatalysis was activated in situ by adding an external iron source to form an in situ photo-Fenton system. Generally, a catalyst sample (30 mg) was added to a reactor with 30 mL phenol solution (10 ppm). Before exposure to light, a certain amount of ferrous sulfate heptahydrate (FeSO_4_·7H_2_O) solid was added into the reactor, then a dark adsorption experiment was carried out for 30 min. The operations of sampling and phenol concentration measurement were the same as described above.

**Detection of hydroxyl radicals (·OH)**: Coumarin was used as a trap to detect the hydroxyl radicals produced in the photocatalytic reaction [34]. In detail, a catalyst sample (30 mg) was added into a reactor containing 30 mL coumarin (1 mM) solution. Before exposure to light, a dark adsorption treatment was carried out for 30 min. During the photocatalytic reaction, samples were taken every 20 min. Finally, the fluorescence spectrum of the solution was measured with a fluorescence spectrometer with a wavelength of 332 nm.

## 3. Results and Discussion

### 3.1. Characterizations of Materials

**Figure 1:**
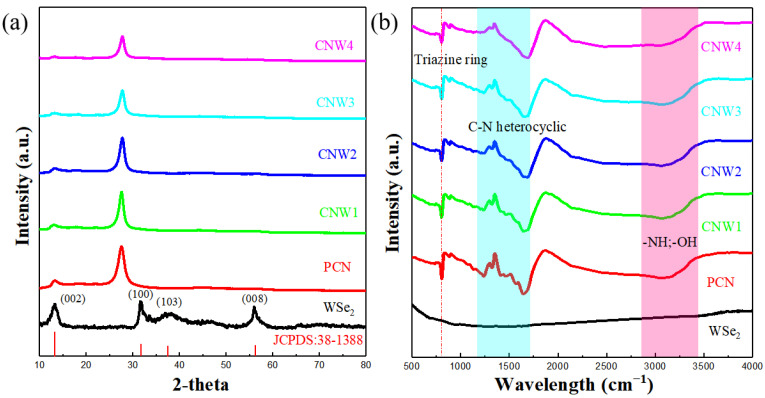
(**a**) XRD spectra and (**b**) FTIR spectra of pure WSe_2_, PCN, CNW1, CNW2, CNW3, and CNW4.

X-ray diffraction was used to investigate the crystal structure of diverse materials. The XRD spectra of pure WSe_2_, PCN, CNW1, CNW2, CNW3, and CNW4 are shown in Figure 1a. The XRD spectrum of the pure WSe_2_ is well matched with its standard card (JCPDS: 38-1388) [35], indicating that WSe_2_ can be synthesized by this method with high purity. The XRD patterns of the CNW materials are nearly identical to PCN, demonstrating that the addition of WSe_2_ does not affect the crystal structure of PCN. There are two characteristic peaks in the spectra of PCN and CNW materials. The peak at 13.1° corresponds to the (100) plane of PCN, representing the repetition of non-planar units. The other peak at 27.5° corresponds to the (002) plane of PCN, which is related to the superimposed reflection of the conjugate plane [36]. In addition, as WSe_2_ loading in CNW composites increases, the intensity of the (100) and (002) peaks gradually weakens. The insignificant peak of WSe_2_ in the CNW materials is due to the low content of WSe_2_.

The FTIR spectra of pure WSe_2_, PCN, CNW1, CNW2, CNW3, and CNW4 are shown in Figure 1b. There is no clear sharp peak in the infrared spectrum of WSe_2_ across the full wavenumber range (500–3500 cm^−1^). The spectrum of CNW composite materials is similar to that of PCN, which indicates that PCN is the primary source of the infrared spectrum signals in CNWs. A tiny peak appearing at 810 cm^−1^ is attributed to the vibration of the triazine unit in PCN and CNW materials. The characteristic peak in the region of 1200–1700 cm^−1^ can be assigned to the C–N heterocyclic ring frame stretching vibration [37,38]. In addition, the characteristic peaks between 3000–3400 cm^−1^ can be assigned to the stretching vibrations of –OH and –NH groups caused by free amino groups in the PCN structure and hydroxyl groups adsorbed on the surface [39].

For the practical study of materials, thermal stability is essential. The TGA plots of the materials were tested in N_2_ atmosphere. All of the produced materials exhibit excellent thermal stability in the 30~500 °C range, as shown in Figure S1. This demonstrates that it is feasible to use CNW materials to treat pollutants in water. The specific surface area of PCN and CNW2 are determined by the nitrogen adsorption–desorption isotherm. As shown in Figure S2, the specific surface area of PCN and CNW2 fitted with the Brunauer–Emmett–Teller (BET) method are 4.61 m^2^/g and 11.83 m^2^/g, respectively. The tiny BET surface area may have little influence on the catalytic activity for H_2_O_2_ evolution or phenol degradation, similar to the results in the literature [30].

**Figure 2:**
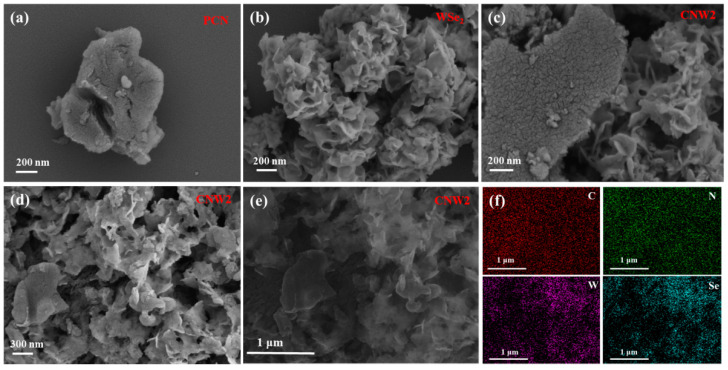
SEM images of (**a**) PCN, (**b**) WSe_2_, and (**c**,**d**) CNW2; (**e**) HAADF-SEM image and (**f**) corresponding EDX elemental mapping images of CNW2.

The morphology of the synthesized samples was obtained by SEM. As shown in Figure 2a, PCN has a large irregular block structure and a relatively smooth surface. In Figure 2b, the synthesized WSe_2_ exhibits a layered petal-like structure self-assembled from ultra-thin nanosheets, consistent with previous studies [40]. Figure 2c,d suggests a few ultra-thin WSe_2_ nanosheets grown on the surface and edges of PCN, which indicates that the WSe_2_ nanosheets were successfully loaded. The WSe_2_ and PCN in the CNW2 are in close contact, which promotes the rapid transfer of photogenerated electrons from the surface of the PCN to the WSe_2_. The distribution of various elements (C, N, W, Se) in CNW2 was studied by element mapping analysis. From the HAADF-SEM and the corresponding EDX elemental mapping images of CNW2 (Figure 2e,f), CNW2 contains four elements, i.e., C, N, W, and Se, and their uniform distribution indicates that CNW nanocomposites were successfully prepared. The SEM image and the corresponding EDX elemental mapping images of PCN, CNW3, and CNW4 are shown in Figure S3.

**Figure 3:**
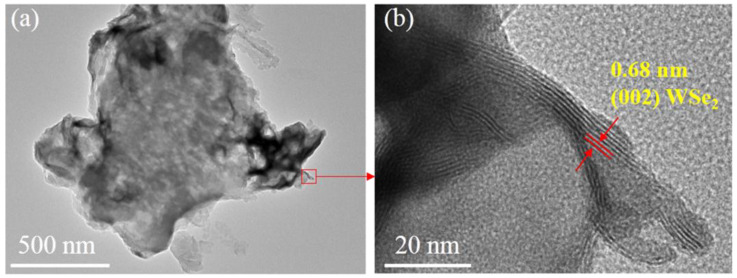
(**a**) TEM image of CNW2 and (**b**) HRTEM image of CNW2.

The micromorphology of PCN and CNW composite materials were further analyzed by TEM and HRTEM (Figure 3 and Figure S4). As shown in Figure S4a,b, there are no lattice fringes locally due to the low crystallinity of PCN. As shown in Figure 3a, WSe_2_ nanosheets are mainly loaded on the edge of g-C_3_N_4_. WSe_2_ exhibits distinct lattice fringes with a fringe spacing of about 0.68 nm, corresponding to the (002) crystal plane of WSe_2_ [40,41]. Moreover, the size of WSe_2_ nanosheets is approximately 30 nm, much smaller than the size of PCN nanosheets. These results reveal that the WSe_2_ nanosheets were successfully loaded onto the PCN, which is consistent with the SEM observations.

**Figure 4:**
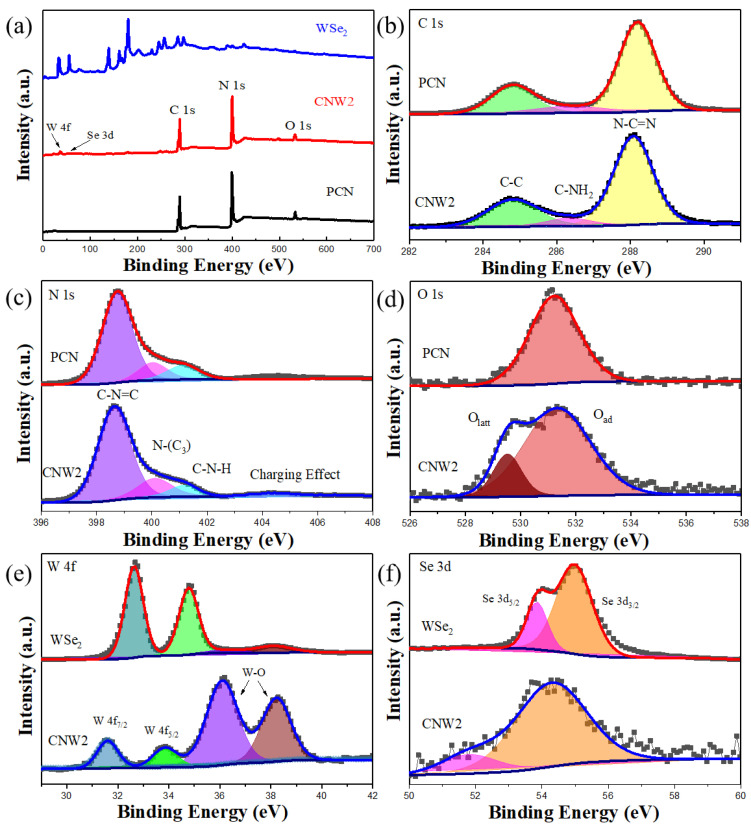
(**a**) XPS survey spectra of WSe_2_, CNW2, and PCN, (**b**) High-resolution XPS C 1s spectra of CNW2 and PCN, (**c**) N 1s spectra of CNW2 and PCN, (**d**) O 1s spectra of CNW2 and PCN, (**e**) W 4f spectra of WSe_2_ and CNW2, and (**f**) Se 3d spectra of WSe_2_ and CNW2.

The XPS survey spectra of WSe_2_, CNW2, and PCN are shown in Figure 4a. The characteristic peaks of the four elements, C, N, W, and Se, can be observed from the XPS spectrum of the CNW2 composite material, which is consistent with the EDX element mapping. The XPS spectrum of CNW2 is similar to that of PCN, because PCN is the major component of CNW2. Because the relative content of WSe_2_ in CNW2 composites is relatively small, the characteristic peaks of W and Se in the XPS spectra of the CNW2 composites are very weak.

Figure 4b–f shows the high-resolution XPS spectra of C 1s, N 1s, O 1s, W 4f, and Se 3d from WSe_2_, CNW2, and PCN. In the XPS spectra of C 1s, the characteristic peak at the binding energy of 284.8 eV corresponds to the extraneous carbon element (C–C bond). Normally, the two peaks at 286.4 and 288.2 eV in PCN are attributed to the C–NH_2_ and N–C=N bonds, respectively [42,43], while in CNW2 composites these two peaks are negatively shifted by 0.1 eV compared to PCN, being located at 286.3 and 288.1 eV, respectively. In Figure 4c, the strongest characteristic peak of PCN located at the binding energy of 398.7 eV indicates the presence of sp^2^ hybrid nitrogen on the aromatic ring of the N atom (C–N=C) [44]. The peak near the binding energy of 400.1 eV is attributed to the tertiary nitrogen N–(C)_3_ group [45]. In addition, two weak peaks at the binding energy of 401.2 and 404.4 eV are attributed to the amino group (C–N–H) and the charging effect in the heterocyclic ring, respectively [42,46]. Similarly, after WSe_2_ loading, the four peaks are all transferred to the lower binding energy positions (398.6, 400.0, 401.1, and 404.3 eV). The XPS spectrum of O 1s is shown in Figure 4d. The peak of CNW2 and PCN at the binding energy of about 532.3 eV is considered to be the adsorbed oxygen species [47]. There is a new peak in the CNW2 composite material appearing at the binding energy of 529.4 eV which can be assigned to the lattice oxygen atom [48], which indicates that the surface of WSe_2_ in the CNW2 composite is slightly oxidized.

As shown in Figure 4e, four typical peaks in WSe_2_ nanosheets are located at the binding energy of 32.5, 34.6, 36.9, and 38.9 eV, respectively. The first two peaks can be attributed to W 4f_7/2_ and W 4f_5/2_ of W^4+^ in pure WSe_2_, respectively. The other two small double peaks can be attributed to the W–O bond, which may be due to the oxidation state of W^6+^ formed by slight oxidation on the surface of the WSe_2_ nanosheets during the synthesis process [49,50,51]. In CNW2 composites, these peaks are all shifted to the lower binding energy positions (31.7, 34.0, 36.2, and 38.4 eV). In addition, the proportion of the W–O peak area of WSe_2_ in CNW2 composites increases due to the loading of WSe_2_ nanosheets on the surface of PCN. Figure 4f shows that the Se 3d spectrum in WSe_2_ can be divided into Se 3d_5/2_ (54.9 eV) and Se 3d_3/2_ (55.8 eV) of the divalent Se ion, which is consistent with previous studies [52,53]. In the CNW2 composite, Se 3d_5/2_ and Se 3d_3/2_ move to positions with binding energies of 51.9 and 54.8 eV, respectively. The changes in the binding energy of these chemical bonds indicate that electrons are transferred from PCN to WSe_2_ nanosheets in the CNW2 samples. Due to the strong Mott–Schottky effect between PCN and WSe_2_, the electron density of WSe_2_ increases, affecting the electronic structure of the two materials.

**Figure 5:**
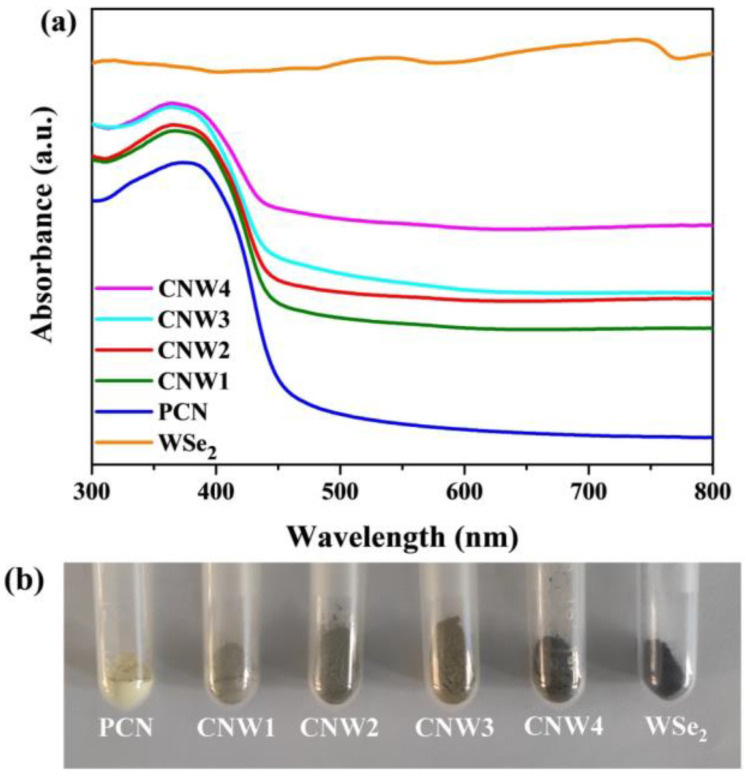
(**a**) UV-vis DRS spectra of PCN, CNW1, CNW2, CNW3, CNW4, and WSe_2_; (**b**) color changes of the various samples.

As shown in Figure 5a, PCN exhibits an absorption band at around 450 nm while pure WSe_2_ has a broad and strong absorption range across the full wavelength (300–800 nm). The visible light capturing ability of CNWs is significantly better than PCN due to the addition of WSe_2_. These changes can be further verified by the changes in the physical appearance of the various samples. As shown in Figure 5b, the increasing content of WSe_2_ in CNW samples causes their color to change from yellow to dark yellow and finally to black. The high light absorption capacity of the CNWs could promote the reaction of photocatalytic H_2_O_2_ production and pollutant degradation.

**Figure 6:**
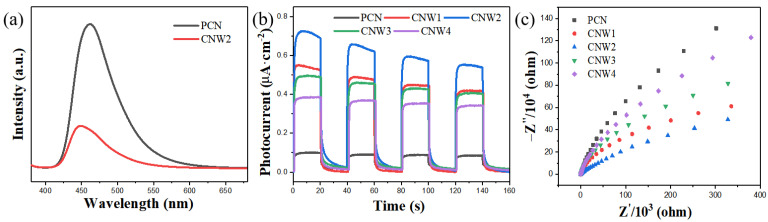
(**a**) PL spectra of PCN and CNW2, (**b**) the transient photocurrent response curves, and (**c**) the electrochemical impedance spectra of PCN, CNW1, CNW2, CNW3, and CNW4.

The PL spectra of PCN and CNW2 are shown in Figure 6a. With an excitation wavelength of 369 nm, a strong emission peak appears at around 462 nm of PCN due to rapid recombination of photogenerated electron-hole pairs in PCN. The fluorescence spectrum intensity of the CNW2 composite emission peak becomes significantly lower than that of PCN after WSe_2_ loading, indicating that WSe_2_ can inhibit the recombination of the electron-hole pairs radiated from PCN. Figure 6b shows the transient photocurrent response curves of the synthesized samples during the typical period of on/off visible light irradiation. The photocurrent is generated immediately after turning on the light, indicating the high photosensitivity and effective space charge separation ability of all samples [54,55]. All of the CNW composites have higher photocurrent response values than PCN, and CNW2 has the highest photocurrent response value with a photocurrent of 0.73 μA cm^−2^. This means that the appropriate content of WSe_2_ can greatly accelerate the separation of charges on the CNW2 sample. The excess black WSe_2_ in CNW composites scatters light and produces a shading effect, reducing the utilization of light. In addition, electrochemical impedance spectroscopy was used to examine the conductivity and interface charge transfer behavior of various samples, as shown in Figure 6c. The semicircular diameter of the CNW2 composite material is substantially smaller than that of PCN, indicating that the interface of the CNW2 sample has better conductivity, contributing to the effective separation and transfer of space charge within the CNW2 material during the photocatalytic reaction [56].

### 3.2. Photocatalytic Performances

**Figure 7:**
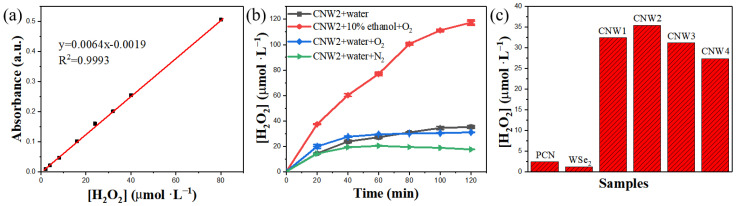
(**a**) Standard curve of the concentration of H_2_O_2_ and its absorbance, (**b**) photocatalytic H_2_O_2_ production profiles on various samples under visible light irradiation, and (**c**) maximum yield of H_2_O_2_ on various samples under visible light irradiation.

The photocatalytic performance of the synthesized materials for H_2_O_2_ production was tested in ultrapure water under visible light. In Figure 7a, the linear coefficient (*R*^2^) of the standard curve is 0.9993, indicating an excellent linear relationship between the concentration of H_2_O_2_ and its absorbance; as such, use of the absorbance to express the content of hydrogen peroxide is credible. The time course of photocatalytic production of H_2_O_2_ under visible light irradiation for the synthesized samples is shown in Figure 7b. The rate of H_2_O_2_ production gradually slows down and finally stabilizes as the reaction progresses, which is caused by the photodecomposition of H_2_O_2_ [57]. As shown in Figure 7b,c, the H_2_O_2_ production of pure WSe_2_ is 0.97 μmol/L. Under visible light for 2 h, the H_2_O_2_ production of PCN is about 2.49 μmol/L, suggesting the low H_2_O_2_ production activity of PCN and the rapid recombination of photogenerated electron-hole pairs [58]. The photocatalytic activity of CNW composite materials is higher than that of PCN and pure WSe_2_, indicating that their combination can significantly improve the production of H_2_O_2_. Among them, CNW2 has the best photocatalytic performance of H_2_O_2_ generation of 35.04 μmol/L, which is almost 14.1 times higher than that of the original g-C_3_N_4_ (2.49 μmol/L). Thus, WSe_2_ is an effective co-catalyst. However, the production of H_2_O_2_ gradually decreases as the amount of WSe_2_ loading in CNW composites is increased up to 7%. The excessive black WSe_2_ in the CNW composite material reduces the utilization of light, thereby reducing the activity of the photocatalytic reaction.

**Figure 8:**
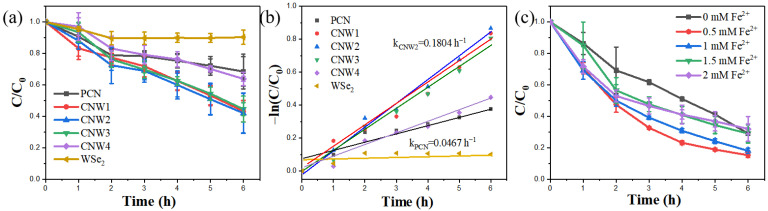
(**a**) Photocatalytic phenol (10 ppm) degradation by the synthesized samples (WSe_2_, PCN, CNW1, CNW2, CNW3, and CNW4) under visible light, (**b**) the corresponding kinetic rate constant k (h^−1^) for photocatalytic degradation of phenol, and (**c**) photocatalytic degradation of phenol (10 ppm) on CNW2 after adding different concentrations of ferrous sulfate heptahydrate under visible light irradiation.

The photocatalytic degradation of phenol (10 ppm) by the synthesized samples was performed under visible light. The visible light source in the multi-channel system had a power of 5 W and the reaction lasted 6 h. As shown in Figure 8a, the degradation efficiency of phenol by pure WSe_2_ is only about 7%. At the same time, PCN shows poor activity, with phenol degrading at a rate of roughly 25% after 6 h. Under similar conditions, the photocatalytic activity of CNWs is higher than that of PCN. The degradation efficiency rates of the CNW4, CNW3, CNW1, and CNW2 samples are 34%, 52%, 61%, and 67%, respectively. Among them, CNW2 has the best catalytic activity in terms of its behavior in photocatalytic H_2_O_2_ production. The WSe_2_ in CNW2 composites can act as a noble metal-free promoter to increase electron mobility and inhibit the recombination of photo-generated electron-hole pairs, thus increasing the catalytic activity of CNW2.

The reaction kinetics of the photocatalytic degradation of phenol were further analyzed using the first order kinetic formula below [59]

(3) $$-\ln\frac{C}{C_{0}}=k\times t$$

where *C*, *C*_0_, *k*, and *t*, represent the concentration of phenol at time *t*, the initial concentration of phenol, the reaction rate constant, and the reaction time, respectively.

The kinetic fitting curves of the synthesized samples are shown in Figure 8b, demonstrating that the order of the photocatalytic reaction rate constant (*k*) of the samples is WSe_2_ < PCN < CNW4 < CNW3 < CNW1 < CNW2. The reaction rate constant of CNW2 increased by about 3.9 times compared to PCN, with respective *k* values of 0.180 h^−1^ and 0.0467 h^−1^.

The CNW2 composite photocatalyst was chosen for the subsequent in situ photo-Fenton degradation of phenol. The photocatalytic degradation of phenol by CNW2 was investigated at various concentrations of ferrous ions (Fe^2+^). In the tests, four different concentrations of Fe^2+^ (0.5, 1.0, 1.5, and 2.0 mM) were selected; the results are shown in Figure 8c. The concentration of Fe^2+^ has a significant influence on the photo-Fenton degradation reaction. Adding a proper concentration of Fe^2+^ can increase the degradation of phenol; at an Fe^2+^ concentration of 0.5 mM, phenol degradation is enhanced to 90%. Fe^2+^ activates the H_2_O_2_ generated by photocatalysis to form an in situ photo-Fenton system by producing more oxidative ·OH and improving photocatalytic degradation efficiency. However, an excessive concentration of Fe^2+^ reduces photocatalytic reaction activity. This may be due to the excessive hydrolysis of Fe^2+^ increasing the acidity of the solution, causing pH to become the dominant factor affecting the photocatalytic reaction. Moreover, compared to the literature (Table S1), the CNW2/Fe^2+^ system has a better phenol degradation rate [60,61,62,63,64,65,66,67,68,69].

### 3.3. Reaction Mechanism

**Figure 9:**
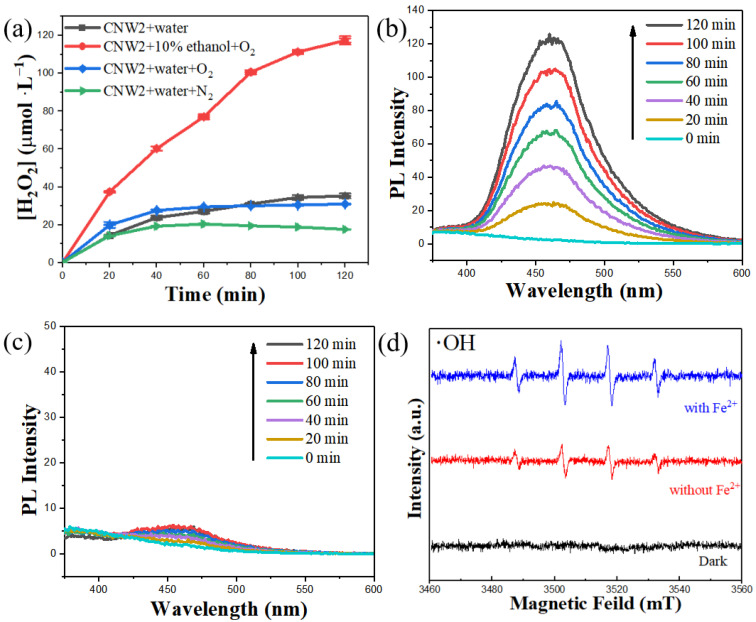
(**a**) Photocatalytic H_2_O_2_ production under different conditions (including CNW2 + water, CNW2 + 10% ethanol + O_2_, and CNW2 + water + O_2_, CNW2 + water + N_2_) for CNW2, fluorescence spectra of 1 mM coumarin solution (**b**) with Fe^2+^ and (**c**) without Fe^2+^ under visible light irradiation; (**d**) EPR spectra of DMPO spin-trapping adducts for CNW2 dispersion with and without Fe^2+^.

Several control experiments were carried out to clarify the production pathway of H_2_O_2_ during the photocatalytic reaction shown in Figure 9a. The results show that the production of H_2_O_2_ can be greatly improved by adding anhydrous ethanol as an electron donor and oxygen gas. When the photocatalytic reaction was carried out in an aqueous ethanol solution with a volume fraction of 10%, the production of H_2_O_2_ increased from 35.04 μmol L^−1^ (pure water) to 117.32 μmol L^−1^, nearly 3.35 times higher than that of pure water, within 2 h of light. When oxygen was injected into pure water, the output of H_2_O_2_ was almost the same as with no gas supplied. At the same time, the production of H_2_O_2_ was slightly suppressed under the nitrogen atmosphere (simulating an anaerobic environment), which indicates that external oxygen has little influence on the photocatalytic reaction. Generally, oxygen is an essential reactant for the generation of hydrogen peroxide whether through a one-step two-electron direct reduction route or a two-step continuous one-electron indirect reduction route [70]. The valence band of g-C_3_N_4_ is about 1.4 eV, and its oxidizing property is sufficient to generate oxygen [54]. Therefore, it should be considered that the holes generated in the valence band of g-C_3_N_4_ in CNW2 can directly oxidize water to generate oxygen. These results indicate that the CNW2 composite material has the potential to generate hydrogen peroxide in an anaerobic environment and can be further used in a variety of other environmental applications, making it a promising photocatalyst.

A coumarin solution was used as a trap to detect the ·OH radicals generated by in situ activation, further confirming the in situ activation of H_2_O_2_. As shown in Figure 9b,c, after adding Fe^2+^, the ·OH radical capture product (7-hydroxycoumarin) has a significant peak at about 460 nm and its fluorescence intensity gradually increases with the progress of the reaction. This shows that as the reaction develops the system continuously generates ·OH radicals, accelerating the degradation of phenol. However, in the absence of Fe^2+^, the characteristic peak of 7-hydroxycoumarin at 460 nm cannot be recognized after 2 h. This indicates that in the absence of Fe^2+^ the amount of ·OH radicals generated by the photocatalytic reaction is almost undetectable. As shown in Figure 9d, the EPR spectra of DMPO spin-trapping adducts for CNW2 dispersion with Fe^2+^ shows stronger signals of ·OH radicals than that without Fe^2+^, which is consistent with the coumarin capture experiments. Fe^2+^ was able to promote the generation of ·OH radicals. The ·OH radical detection experiment further proved that in combination with Fe^2+^, the CNW2 photocatalyst could be used to construct an in situ photo-Fenton system for direct phenol degradation without additional H_2_O_2_.

## 4. Conclusions

In this work, several WSe_2_/g-C_3_N_4_ photocatalysts with different composite ratios were successfully synthesized by a hydrothermal and calcination method, then characterized by XRD, FTIR, SEM, and TEM. The synthesized samples were applied to photocatalytic H_2_O_2_ production and photocatalytic degradation of phenol. Among them, CNW2 with 7% loading of WSe_2_ displayed the greatest photocatalytic performance with H_2_O_2_ production of 35.04 μmol/L in two hours, which is about 14.1 times that of PCN. Meanwhile, the phenol degradation efficiency of CNW2 reached 67%, 42% higher than that of PCN. By constructing an in situ photo-Fenton reaction, the addition of 0.5 mM Fe^2+^ was able to further promote the photocatalytic degradation of phenol to 90%. However, there are aspects that can be further studied, such as in the removal of other organic pollutants, the purification of actual polluted water, environmental toxicity, etc. Overall, this work provides new insights for developing new materials for H_2_O_2_ production in pure water and for in situ photo-Fenton reaction to degrade organic pollutants.

## Data Availability

Not applicable.

## Supplementary Materials

The following supporting information can be downloaded at: https://www.mdpi.com/article/10.3390/nano12183089/s1. Figure S1. TGA plots of PCN, CNW1, CNW2, CNW3 and CNW4. Figure S2. Nitrogen adsorption-desorption isotherm of PCN and CNW2. Figure S3. SEM image and the corresponding EDX elemental mapping images of PCN, CNW3 and CNW4. Figure S4. (a,b) TEM and HRTEM images of PCN, (c,d) TEM and HRTEM images of CNW3, (e,f) TEM and HRTEM images of CNW4. Table S1. Comparison of phenol degradation performance under visible light.
