# Supplementary material for: WSe2/g-C3N4 for an In Situ Photocatalytic Fenton-like System in Phenol Degradation
Source: Nanomaterials (Basel). 2022 Sep 6;12(18):3089. doi: 10.3390/nano12183089 (PMC9501952; doi:10.3390/nano12183089)
Supplement: Supplementary file 1 [file nanomaterials-12-03089-s001.zip › nanomaterials-1881933-supplementary.pdf]

Supporting Information

$WSe_2/g\text{-}C_3N_4$ for an In Situ Photocatalytic Fenton-like System in Phenol Degradation

Li Tan [1,2], Yiming Chen [1,2], Didi Li [1,2], Shaobin Wang [3,*] and Zhimin Ao [1,2,4,*]

[1] Guangdong-Hong Kong-Macao Joint Laboratory for Contaminants Exposure and Health, Guangzhou Key Laboratory Environmental Catalysis and Pollution Control, Institute of Environmental Health and Pollution Control, Guangdong University of Technology, Guangzhou 510006, China

[2] Guangdong Key Laboratory of Environmental Catalysis and Health Risk Control, Key Laboratory for City Cluster Environmental Safety and Green Development of the Ministry of Education, School of Environmental Science and Engineering, Guangdong University of Technology, Guangzhou 510006, China

[3] School of Chemical Engineering and Advanced Materials, The University of Adelaide, Adelaide, SA 5005, Australia

[4] Advanced Interdisciplinary Institute of Environment and Ecology, Beijing Normal University, Zhuhai 519087, China

* Correspondence: shaobin.wang@adelaide.edu.au (S.W.); zhimin.ao@bnu.edu.cn (Z.A.)

Figure S1

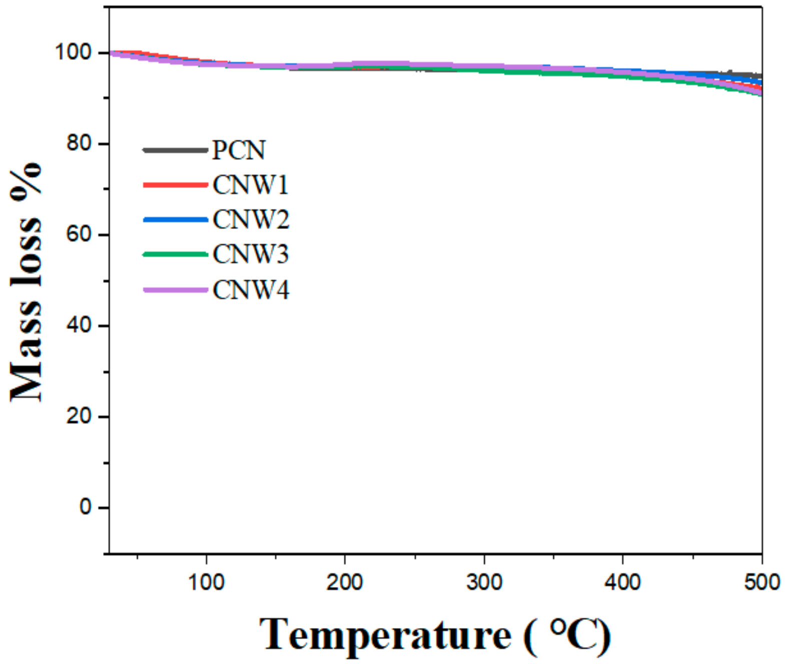

Figure S1. TGA plots of PCN, CNW1, CNW2, CNW3 and CNW4.

Figure S2

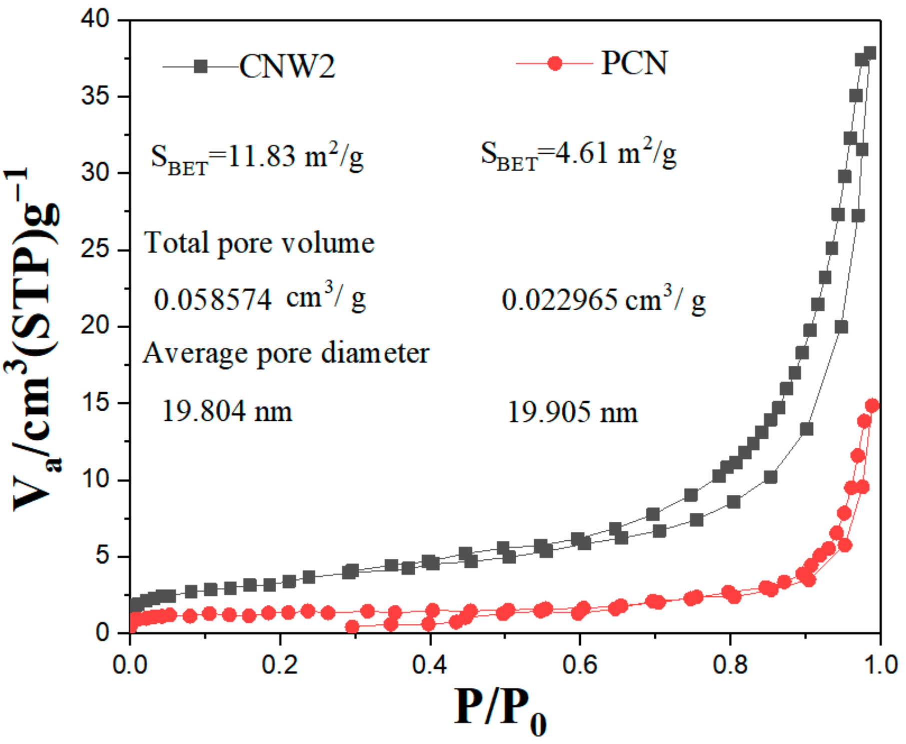

Figure S2. Nitrogen adsorption-desorption isotherm of PCN and CNW2.

Figure S3

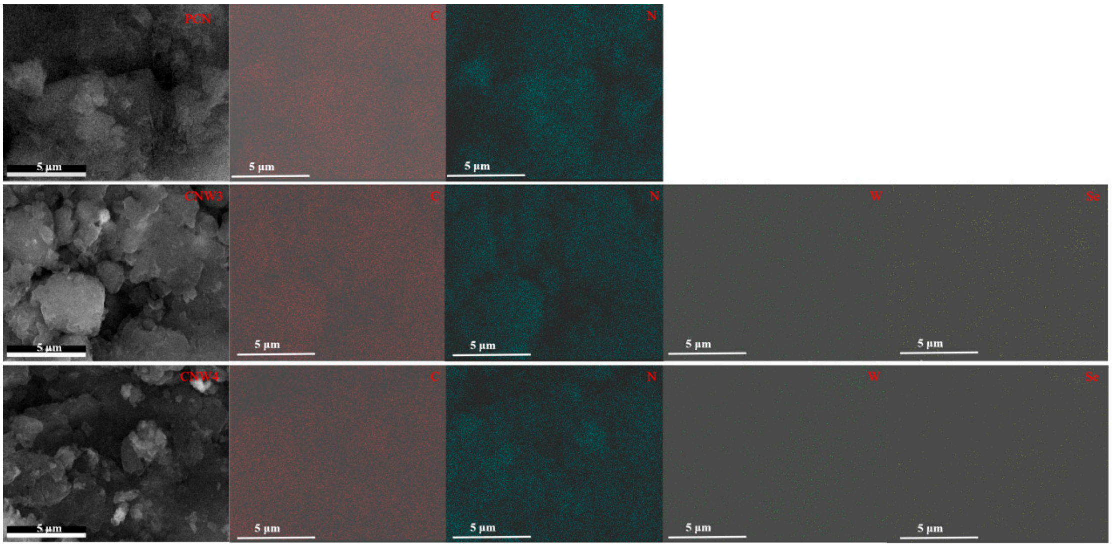

Figure S3. SEM image and the corresponding EDX elemental mapping images of PCN, CNW3 and CNW4.

Figure S4

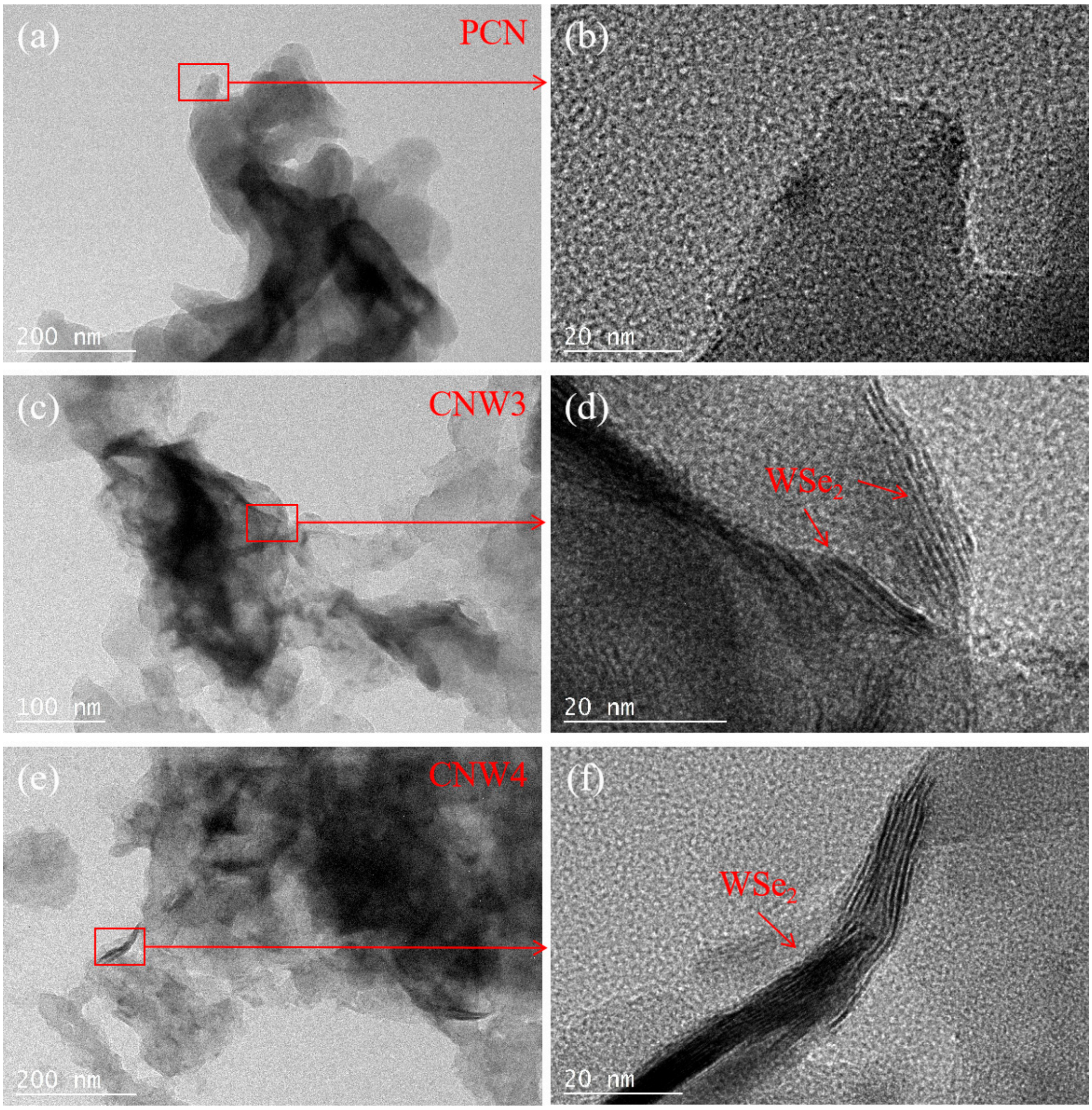

Figure S4. (**a**,**b**) TEM and HRTEM images of PCN, (**c**,**d**) TEM and HRTEM images of CNW3, (**e**,**f**) TEM and HRTEM images of CNW4.

Table S1. Comparison of phenol degradation performance under visible light.

Sample	Usage amount	Phenol Concentration	Degradation	Reference
CNW2	1 g/L	10 mg/L	90%	This work
5%C/TiO_2	2 g/L	10 mg/L	< 50%	[1]
FSM-GO	1 g/L	20 mg/L	85%	[2]
Fe^{2+}/H_2O_2/CuO	100 g/L	--	86.77%	[3]
MCN	1 g/L	50 ppm	40%	[4]
$BiVO_4$/SiO_2/GO	1 g/L	20 ppm	92%	[5]
AC-TiO_2	0.5 g/L	50 mg/L	55%	[6]
CS B-TiO_2	0.5 g/L	40 ppm	70.20%	[7]
CAPS-MWCNT-4	--	5 mg/L	90.6%	[8]
MCN	1 g/L	50 ppm	74%	[9]
TiO_2@Fe	1.87 g/L	10 mg/L	33%	[10]

Reference

1. Zhang, J.; Zheng, Y.; Ma, M.; Li, H. Structural Characterization and Photocatalytic Activity of Synthesized Carbon Modified TiO_2 for Phenol Degradation. *J. Wuhan Univ. Technol.-Mat. Sci. Edit.* **2020**, *35*, 535-540, doi:10.1007/s11595-020-2290-9.
2. Vasallo-Antonio, R.; Peña-Bahamonde, J.; Susman, M.D.; Ballesteros, F.C.; Rodrigues, D.F. Design and performance of Fe_3O_4@SiO_2/MoO_3/polydopamine-graphene oxide composites for visible light photocatalysis. *Emergent Mater.* **2021**, *4*, 1425-1439, doi:10.1007/s42247-020-00142-w.
3. Firdharini, C.; Setyaningtyas, T.; Riyani, K. Comparative study of Fe^{2+}/H_2O_2/CuO/Vis and Fe^{2+}/H_2O_2/CuO for phenol removal in batik wastewater under visible light irradiation. *J. Phys.: Conf. Ser.* **2021**, *1918*, 032004, doi:10.1088/1742-6596/1918/3/032004.
4. Lee, S.C.; Lintang, H.O.; Endud, S.; Yuliati, L. Highly Active Mesoporous Carbon Nitride for Removal of Aromatic Organic Pollutants under Visible Light Irradiation. *Adv. Mater. Res.* **2014**, *925*, 130-134, doi:10.4028/www.scientific.net/AMR.925.130.
5. Trinh, D.T.T.; Channei, D.; Nakaruk, A.; Khanitchaidecha, W. New insight into the photocatalytic degradation of organic pollutant over $BiVO_4$/SiO_2/GO nanocomposite. *Sci. Rep.* **2021**, *11*, 4620, doi:10.1038/s41598-021-84323-5.
6. Asencios, Y.J.O.; Lourenço, V.S.; Carvalho, W.A. Removal of phenol in seawater by heterogeneous photocatalysis using activated carbon materials modified with TiO_2. *Catal. Today* **2022**, *388-389*, 247-258, doi:10.1016/j.cattod.2020.06.064.
7. Nawaz, R.; Haider, S.; Ullah, H.; Akhtar, M.S.; Khan, S.; Junaid, M.; Khan, N. Optimized remediation of treated agro-industrial effluent using visible light-responsive core-shell structured black TiO_2 photocatalyst. *J. Environ. Chem. Eng.* **2022**, *10*, 106968, doi:10.1016/j.jece.2021.106968.
8. Wu, G.; Xing, W. Facile Preparation of Semiconductor Silver Phosphate Loaded on Multi-walled Carbon Nanotube Surface and Its Enhanced Catalytic Performance. *J. Inorg. Organomet. P.* **2018**, *29*, 617-627, doi:10.1007/s10904-018-1036-z.
9. Lee, S.C.; Lintang, H.O.; Yuliati, L. A urea precursor to synthesize carbon nitride with mesoporosity for enhanced activity in the photocatalytic removal of phenol. *Chem. Asian J.* **2012**, *7*, 2139-2144, doi:10.1002/asia.201200383.
10. Hu, J.Y.; Tian, K.; Jiang, H. Improvement of phenol photodegradation efficiency by a combined g-C_3N_4/Fe(III)/persulfate system. *Chemosphere* **2016**, *148*, 34-40, doi:10.1016/j.chemosphere.2016.01.002.
